# Process-Induced Distortions Characterization of MBWK Fabric Reinforced Composite Helmet Shell

**DOI:** 10.3390/ma13132983

**Published:** 2020-07-04

**Authors:** He Xiang, Yaming Jiang, Yexiong Qi, Jialu Li

**Affiliations:** 1 School of Textile Science and Engineering, Tiangong University, Tianjin 300387, China; xianghe68@126.com (H.X.); qiyexiong@tiangong.edu.cn (Y.Q.); lijialu@tjpu.edu.cn (J.L.); 2 Key Laboratory of Advanced Textile Composite of Ministry of Education, Tiangong University, Tianjin 300387, China

**Keywords:** 3D thin shell textile composites, 3D laser scanning machine, process-induced distortions, non-contact measurement, feature distance

## Abstract

In order to characterize the process-induced distortions of 3D thin shell composites with complex shape, the multilayered biaxial weft knitted (MBWK) fabric reinforced high-performance composite helmet was selected as the research object, and the 3D laser scanning machine was used to scan the helmet surface, then the 3D scanning data was compared with the CAD model to evaluate the deformation. The results and discussion indicated that the conventional method was workable, but the speed of convergence was slow and the calculation results were easy to drop into local optimization. According to detailed analysis, a measurement method focusing on the principle of “Feature Distance” was developed. The measurement results shown that this method can not only give accurate results, but also reduce working procedure and greatly save the computing resources, which is proved to be a feasible approach for the deformation measurement foundation of 3D thin shell textile composites.

## 1. Introduction

Since the fiber reinforced composites have excellent fatigue resistance, high strength-to-weight and stiffness-to-weight ratios, they have been widely employed in various fields [1,2,3]. As one of the structures in composite engineering, thin shell structure is commonly used in advanced equipment, such as helmet, oxygen mask and unmanned aerial vehicle, etc. [4,5]. However, the residual stress is an inevitable problem during the manufacturing process of composites, which are caused by the mismatch of the coefficients of thermal expansion (CTE) between reinforced fiber and matrix, the shrinkage in the resin polymerization reaction during the curing process, and fiber reorientation during the 3D shape forming process [6,7,8,9,10,11] Additionally, a number of literatures implied that residual stresses may cause composite delamination and matrix fracture, reduce the failure strength, and influence the performance and service life of composite materials. Additionally, the process-induced distortions (PIDs) may affect the structural integrity even lead composites to fail [12,13]. Just due to these problems, the application of thin shell composites is seriously restricted. Therefore, it is meaningful to measure and evaluate the distortion of composite materials with efficient technology.

In recent years, with the development of three-dimensional laser scanning technology, it has developed rapidly in the field of composites quality detection, such as deformation monitoring, dimensional evaluation, and surface defects characterization, etc. [14,15,16]. The analysis results can be employed to die compensation, and process adaptation [17]. Moreover, three-dimensional digital detection is one of the most important key factors of controlling the composite product quality, especially in terms of accurate size control.

Measuring and characterizing the deformations of 3D structural composites have been studied by many researchers [18,19,20,21]. Filippatos et al. [22] used an optical marker recognition system to determine the thickness variation of the composite rotors, for verifying the simulation analysis of the gradual damage behaviour of composite rotors. Xu et al. [23] investigated the deformation of large-scale composite tunnel structure with terrestrial laser scanning (TLS) technology, which could be applied in health monitoring. According to Yang et al. [24], a series of statistical experiments on composite structures under monotonic loads based on TLS measurement were conducted to investigate the deformation behavior of the arch structure. The measurement results also had been applied to build finite element modeling for composite arched structures deformation simulation [25]. Although the existing works made a tremendous contribution to the composite deformation measurement, the tested samples were composed of several planes like L beam, or continuous same curved cross-section such as tunnel, arch, Z shape, U shape, etc. The matter of whether the methods applied to the complex structure with different curved cross-section was not mentioned, such as helmet, oxygen mask, and propeller, etc. which are difficult to find the geometrical center point, centerline, or datum plane when the local surface deformed [26]. According to the literatures mentioned above, the calculation speed is slow, and the accuracy is slow. On the other hand, it is hard to integrate the scanned model and design model into the common coordinating system with the different original points [27].

This paper investigates the process-induced distortions of the MBWK fabric reinforced composite helmet. By comparing the point cloud data with CAD model, the result indicates that it is sometimes difficult to avoid local optima when using traditional iterative closest point (ICP) algorithm to register the point cloud data with the designed CAD model. And a novel measuring strategy based on the “feature distance” is developed, it can characterize the distortion of the composites by the size of the critical parts, which calculate the deformation by scalar rather than vector. This method takes up less computing resources and can achieve good results, in addition, there is no registration convergence problem in this method. Combining with the previous method, it can be used to detect the deformation of complex curved composite structure.

## 2. Composite Materials and Manufacturing Process 

### 2.1. Preparation of Raw Materials

**Figure 1:**
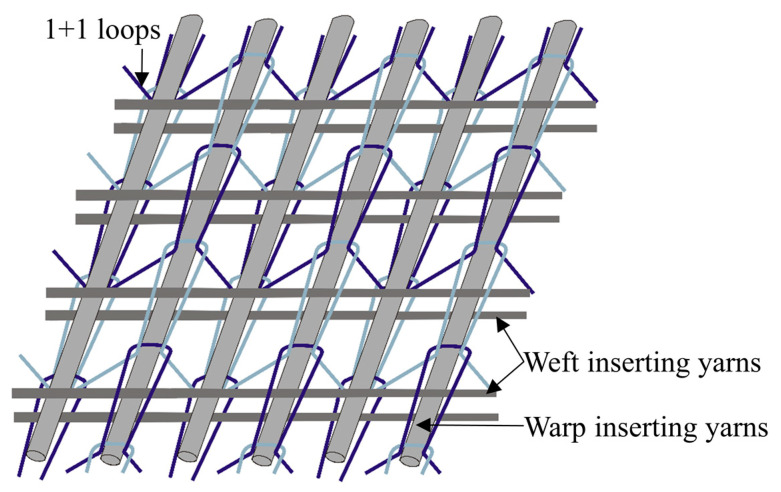
MBWK structure with three layers of inserting yarns.

In this study, the multilayered biaxial weft knitted (MBWK) fabric is selected as reinforcement. The MBWK fabrics, a member in the non-crimp fabrics (NCFs) family, of good formability on curved molding surfaces and of high property-to-cost ratio, can behave as reinforcements for the composite plate and shell structures [28]. The three-layer-connected biaxial weft knitted fabric is chosen for this experiment, as the structure shown in Figure 1, where the stitching system structure is 1 + 1 rib, which has good stretch property and can provide adequate space for inserting yarns shearing [29,30,31], and the inserting yarns are parallel and straight, hence the utilization rate of high performance fiber is over 90% [32]. In addition, the various type of inserting yarns hybrid ratio lend the MBWK fabric flexible designability [33]. Literatures show that the MBWK fabrics are widely applied in the fields of personal protective equipment, wind power, and automobile industry [33,34]. 

In order to build a helmet shell for high-speed impact resistance, three kinds of high performance fibers are selected as inserting yarns, which are aramid fiber (Kevlar 49), carbon fiber (T-300), and ultra-high molecular weight polyethylene (UHWPE). Raw materials’ specification is shown in Table 1. And all the stitch yarns are polyester (PET DTY). 

**Figure 2:**
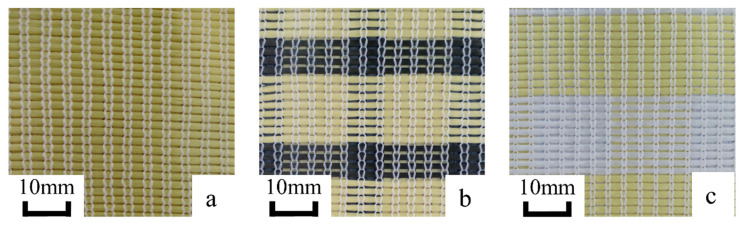
Three types MBWK fabrics: (**a**) Aramid; (**b**) Aramid/carbon; (**c**) Aramid/UHMWPE.

The parameters of three types MBWK fabrics with different hybrid ratio were listed in Table 2. The samples are presented in Figure 2.

The resins are vinyl ester (VE), unsaturated polyester (UP) and epoxy (EP), respectively, as shown in Table 3.

### 2.2. Sample Preparation

**Figure 3:**
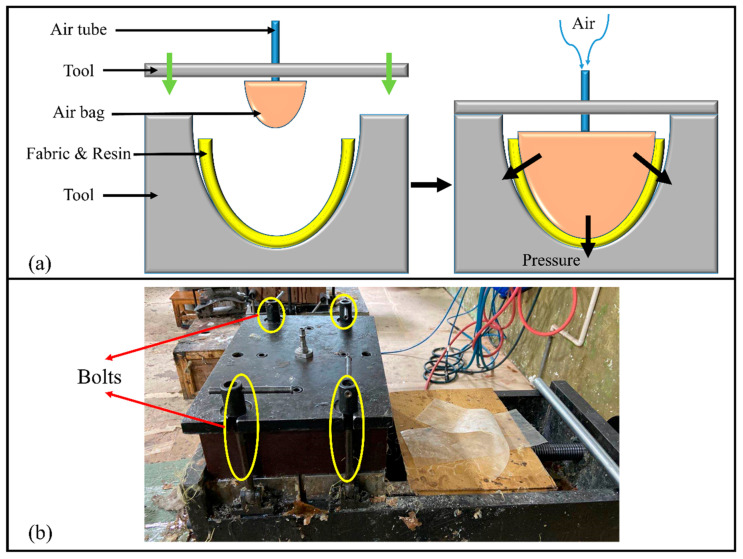
Sample preparing process: (**a**) A schematic diagram of air bag compression molding process; (**b**) Actual molding set-up.

The composite samples are prepared by the air bag compression molding process, which is suitable for the small or medium-sized products with complex structure and smooth surface, as shown in Figure 3a. 

In the preparing process includes firstly the MBWK fabric is put into the tool, the size of the fabric sample is 50 × 50 cm, then the resin is applied evenly in the fabrics by manual lay-up method, the weight of mixed resin for each sample is 110 g. After closing the tool and screwing down bolts (the calibration of the tools’ position also depends on the bolts), as shown in Figure 3b, the air bag is inflated with 0.3 MPa pressure for four hours under room temperature. Afterwards, the sample is demolded after releasing the air and opening the tool, if there are no dry spots on the surface, this composite shell is considered to be a qualified one. Finally, the trimming process is applied to cutting the sample’s extra parts. In this experiment the trimming machine is Prima Rapido-5^®^ 3D laser cutting machine, all the trimming path is predesigned and automatically executed by the machine, the laser power for cutting sample is 3.5 kW. The symbolizations of sample composite shells are listed in Table 4.

## 3. Data Extraction and Analysis

### 3.1. Helmet Molding and Surface Scanning

**Figure 4:**
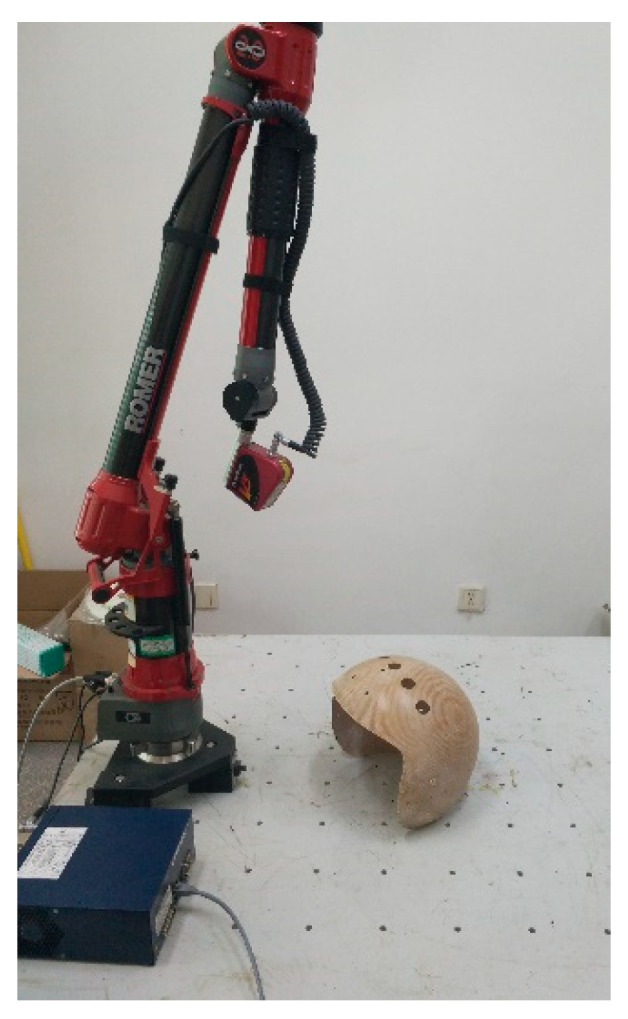
Measurement step of the helmets using a 3D laser scanning machine.

In order to release the residual stresses and spring-back as fully as possible, the helmets are relaxed for about one week or so, then the surfaces are scanned by 3D measuring arm Romer^®^ with laser scanner Scanworks^®^ V3, the measuring arm is fixed on optical table, as illustrated in Figure 4. The laser system technical data is presented in Table 5. During the scanning process, the calibration of the sensor and the collection of the point cloud data are accomplished by PC-DIMIS software. Then, the scanned point cloud data are imported into Geomagic^®^ software (3D Systems^®^) in bin format to optimization and building.

### 3.2. Conventional Analysis Method based on ICP Algorithm

The point cloud and the CAD model should be aligned in the same coordinate system firstly, which is known as registration. The most commonly used method of registration is the Iterative Closest Point (ICP) algorithm, the ICP algorithm aims to minimize the difference between a point cloud and some reference surface, by minimizing the square errors between the corresponding entities, to find the transformation between them [35,36,37]. More specifically, there are two corresponding point sets:

$$X=\left\{x_{1},\cdots ,x_{n}\right\}$$

$$Y=\left\{y_{1},\cdots ,y_{n}\right\}$$

the goal of the ICP algorithm is to find the translation ***t*** and rotation ***R*** that minimizes the sum of the squared error:

$$E\left(R,t\right)=\frac{1}{n}\overset{n}{\underset{i=1}{\sum }}\|x_{i}-Ry_{i}-t{\|}^{2}$$

where ***x_i_*** and ***y_i_*** are corresponding points.

Generally, the procedure for the initial scanning model preprocess is as follows: combine point clouds to one object, filter the disconnected components, and then clean noise. The coordinate systems of the design model and the scanned model are not consistent when they are created [36]. Therefore, the initial positions of the two models are often scattered after importing the scanned model and CAD model into Geomagic^®^. The ICP algorithm requires that the initial positions between the test and reference models are close enough, since the point cloud and the CAD model has been in the different coordinate system when they are built, it is necessary to narrow the primary direction deflection and location deviation of the measured data and CAD model, otherwise it may not be able to obtain good convergence results. Hence, it is necessary to have a coarse registration before fine registration with ICP algorithm. Thus, it is essential to manually move the two models as close as possible in the same coordinate system for coarse registration, then align the two models with the Best Fit Alignment tool automatically, which is a fine registration step based on ICP algorithm [38].

**Figure 5:**
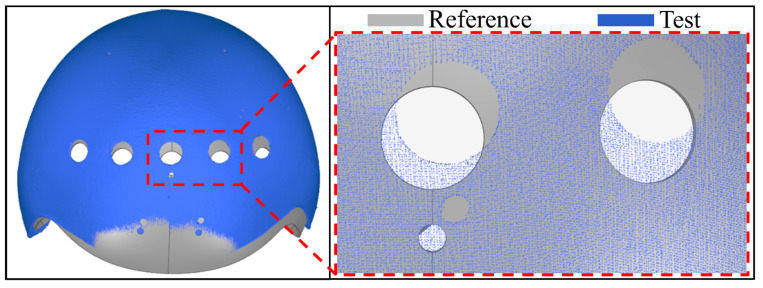
Alignment result based on ICP algorithm.

However, in this study the scanned model and CAD model still have different degrees of malposition after alignment. As shown in Figure 5, the assembly holes’ locations obviously illustrate the deviation between the reference model and test model, which can lead to theoretical errors in the deformation evaluation results. The reason is that the warpage occurs to helmet samples to some degrees after being cured completely, which makes it impossible to obtain a point cloud with the same shape as the CAD model, and the geometric center has changed, which increases the difficulty of alignment [36]. On the other hand, the alignment method based on ICP algorithm is easy to drop into local optimum [26,36,39], the local optimums typically do not correspond to the correct result, because there is not enough geometry shape to restrict the sliding between two partial shapes. Because the ICP assumes that one point set is a subset of the other. When this assumption is not valid, false matches are created which negatively influences the convergence of the ICP to the correct solution [40]. Accordingly, the alignment results can not be used to obtain or evaluate the samples’ deformation.

### 3.3. Analysis Based on Feature Distance

Modern advanced helmets can both provide protection also other intelligent functions through assembling different sensors [41,42]. Therefore, the dimension of the helmet and the deviation of the assembly holes’ location will directly affect the accuracy of the helmet system. In order to characterize the overall deformation tendencies of the helmets, seven circular holes in the CAD model are selected as research objects according to the critical assembly criteria, and center points are obtained by feature extracting. Then, seven corresponding circular holes are found in the point cloud model, and their center coordinates are extracted. The Euclidean distance between the center of the circle is used to investigate the deformation of the helmets:

(1) $$d=\sqrt{{\left(x_{1}-x_{2}\right)}^{2}+{\left(y_{1}-y_{2}\right)}^{2}+{\left(z_{1}-z_{2}\right)}^{2}}$$

where the d refers to the distance between any two points in three dimensional coordinate like point 1 (x1, y1, z1) and point 2 (x2, y2, z2).

**Figure 6:**
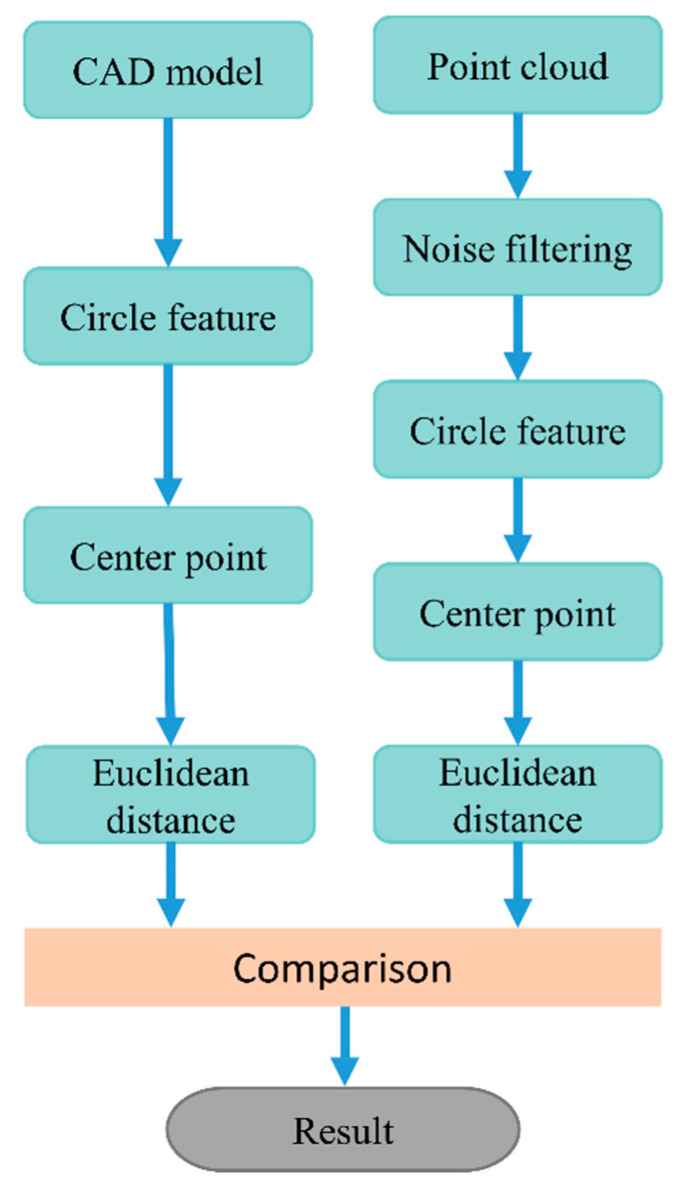
Work-flow of “feature distance” strategy.

**Figure 7:**
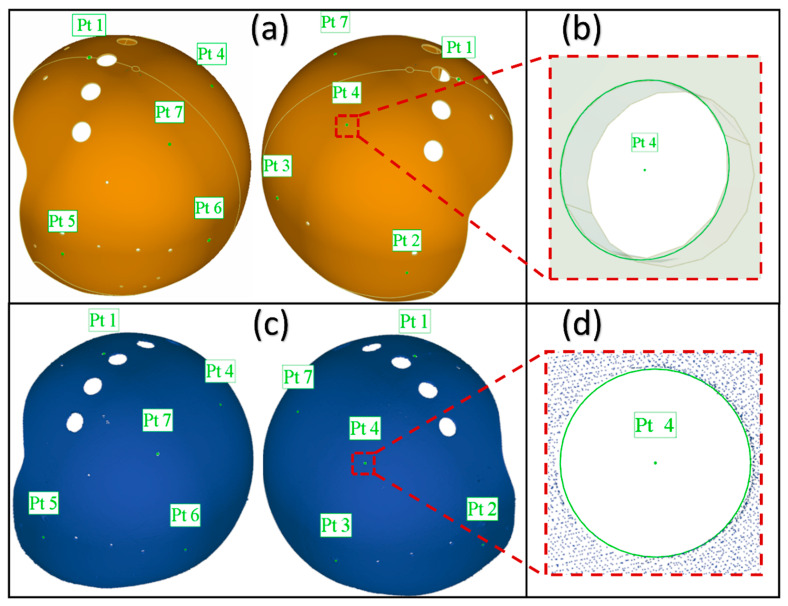
The central points of design model and point cloud model: (**a**) The location of central points in CAD model; (**b**) Central point obtaining in CAD model; (**c**) The location of central points in point cloud model; (**d**) Central point obtaining in point cloud model.

**Figure 8:**
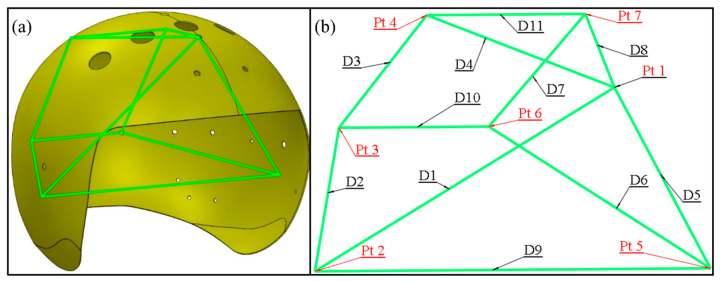
Feature points and feature distance: (**a**) The feature of CAD model; (**b**) Feature note.

This paper puts forward an evaluation strategy called “feature distance”, the progress is presented in Figure 6. Firstly, the seven assembly holes locate on the top, rear and both sides of the helmet in the CAD model (as shown in Figure 7a) have been selected. The “feature” function of Geomagic^®^ is used to build circular feature on the model surface as presented in Figure 7b. After that the “point location” function of “analysis” tool is used to export the coordinate of the circle center, and number the distance between center points as shown in Figure 8 and Table 6. Then the distances between these central points are calculated accord to the Euclidean distance formula.

During the filtration process: the type of noise reduction is “free-form shapes”, the Smoothness Level is 2 and the Iterations value is 5. After filtering the noise of the cloud data, the “features” function is used to build circle features in the assembly holes of the scanned model on the common location with the CAD model, as shown in Figure 7d, and obtain the coordinates of the circle central points. Afterwards, number the seven central points with the same sequence and calculate the Euclidean distance according to the Table 6.

Finally, use the 11 “feature distance” in each 3D point clouds to compare with the “feature distance” in the CAD model in common code. The absolute values sum of the deviation in each scanned model are used to evaluate the deformation of each helmet totally.

## 4. Results and Discussion

**Figure 9:**
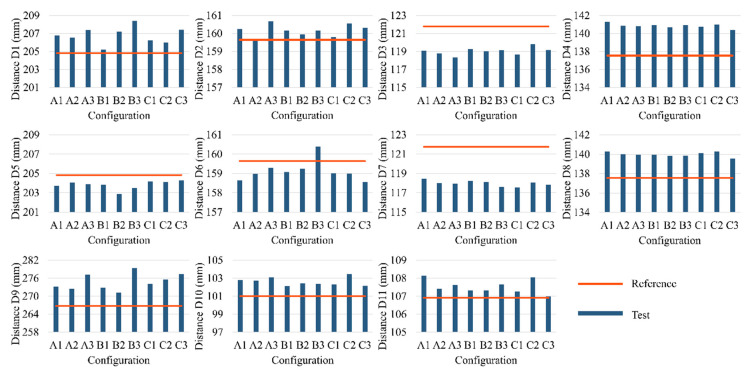
Feature distance results.

As mentioned previously, the measurement results based on the “feature distance” method of the nine helmets are shown in Figure 9, the detailed data is presented in Appendix A. The eleven feature distances of CAD model are used as reference line, each reference line is used to compare with the nine testing results for evaluating the deviations. The results demonstrate that all the helmets made by different fibers and resins deformed obviously but at different levels.

**Figure 10:**
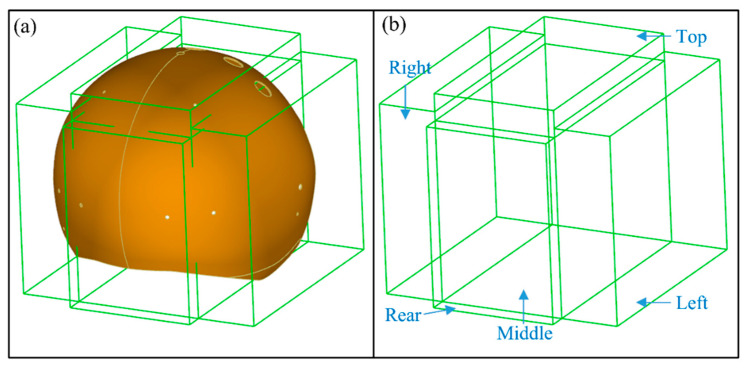
Division of the helmet: (**a**) Dividing the helmet into five areas; (**b**) Names of five areas.

**Figure 11:**
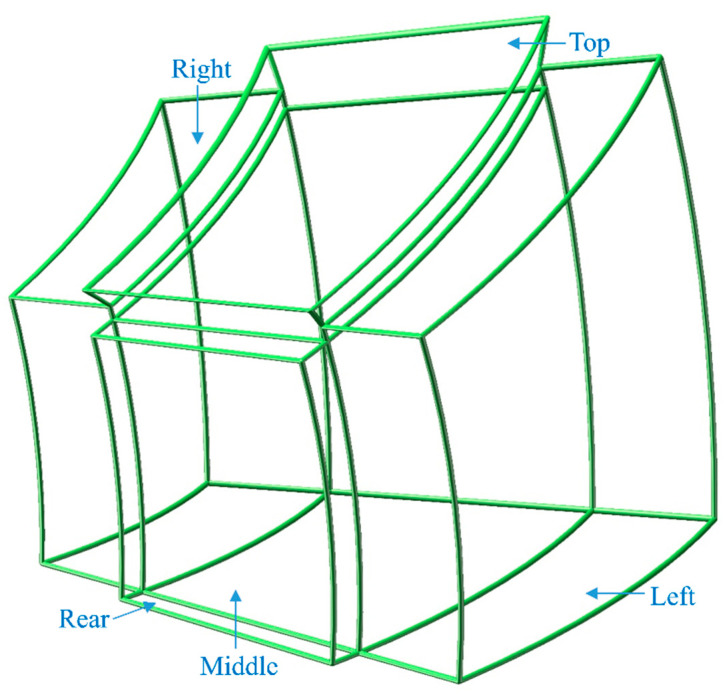
The distortion tendencies of five areas.

In order to facilitate the explanation of PIDs, the helmet is divided into five parts, i.e., left, right, top, middle and rear, as shown in Figure 10. It can be seen that the size of left area is represented by the feature distance D1 and D2, combining the Figure 8b with Figure 9, the measuring results show that D1 and D2 of the nine helmets become larger on an average of 1.961 mm and 0.525 mm respectively, which indicate that the size of the nine helmets’ left areas has an increasing trend. Likewise, the results of D4 and D8 show an average length increase of 3.309 mm and 2.429 mm, suggesting that the helmets’ top areas become larger. The measurements of D5 and D6, however, indicate that the majority of helmets’ right areas become narrower, the length of D5 and D6 show an average narrowing of 1.004 mm and 0.665 mm, and the D3 and D7 values follow the same law, which also show a decrease by an average 2.740 mm and 3.789 mm length decrease, which demonstrate the reduction trend of the sizes of helmets’ rear area. But D6 value of sample B3 is an exception, which becomes wider. As seen from the results of D9, D10 and D11, the top, middle and rear areas have the phenomenon of lateral expansion and the length grow by an average of 8.077 mm, 1.602 mm and 0.630 mm respectively. All the deformations are expressed by the five parts as shown in Figure 11. It can be seen from the results that the sizes of the helmets’ top area are basically consistent with the requirements of the design, and the rear parts have some deviations but small, which are caused by spring-back after demoulding. In addition, the locations of helmets’ maximum spring-back appear in the lateral bottom areas, due to their free boundary with low restriction.

**Figure 12:**
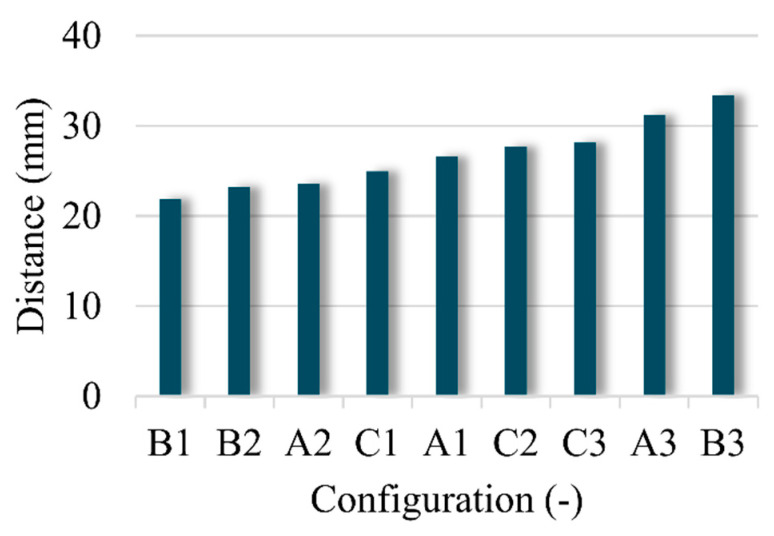
Total deformation results of nine helmets.

Furthermore, the total deformation result of each helmet can be achieved by summing up the absolute values of the deviations between the measured and standard feature distance of each helmet, as shown in Figure 12. According to the final evaluation results, the helmet made of aramid and UP has the smallest deformation while the helmet made of aramid/UHMWPE and UP has the largest deformation after using the same processing parameters. From the results of A3, B3 and C3, the helmet made of UHMWPE as reinforcement is easier to deform, because of its low modulus and weak resin bound ability.

## 5. Conclusions

In this paper, 3D laser scan technology was adopted to investigate the process-induced distortion of complex surface thin shell composites. The method based on “feature distance” was proposed to characterize the deviation of the local feature, which could reflect the integral deformation of the helmets.

It was shown that the ICP algorithm is difficult to register the design model with actual model when the amount of point data is huge or initial position is unreasonable, easily appear some problems in the registration process such as non-convergence and local optimum. However, with the development of laser scanning technology, there are increasing amounts of point cloud data, and that makes it harder to register the reference model with measured model with complex curved structure by traditional ICP algorithm. However, the “feature distance” method can avoid the theoretical mistakes resulted from the ICP algorithm, furthermore, it can omit the registration process. Meanwhile, one model at a time of procedure can save much computation resource.

The results of the nine helmets deformation based on the “feature distance” evaluation strategy shown that the distance between left and right sides become larger are the main deformation characteristic of all the helmets, the total distortion of the aramid/carbon hybrid UP helmet is smallest, the aramid/UHMWPE hybrid EP helmet has the maximum integral curing deformation. Thereby, the helmet made by aramid/carbon hybrid UP has better dimensional accuracy with the same processing parameter. This method is feasible and effective for deformation measurement and dimension evaluation of complex thin shell composites.

Herein, the evaluation method based on the distance among the critical positions to characterize the deformation of 3D thin shell textile composites and combined with laser scan technology was put forward. The method is convenient, rapid, accurate, with suitable for the complex shelled composites intelligent deformation monitor. In addition, it provides a reference for improving the efficiency of resin matrix composite quality control and deformation inspection.

## Figures and Tables

**Table 1:** Specification of inserting yarns.

Type	Density	Tensile Strength	Tensile Modulus	CTE
	/(g·cm^−3^)	/GPa	/GPa	/10^−6^ ℃
Carbon	1.76	3.53	230	−0.54
Aramid	1.44	3.00	112	−2.7
UHMWPE	0.97	3.20	99	−12

**Table 2:** Parameters of three types MBWK fabrics.

Type	Fineness of Stitch Yarns (Polyester)	Weft Yarns’ Composition	Warp Yarns’ Composition	Thickness
Aramid	75D × 2	Aramid 100%	Aramid 100%	1.0 mm
Aramid/Carbon	75D × 2	Aramid: Carbon 8: 4	Aramid: Carbon 8: 4	0.9 mm
Aramid/UHMWPE	75D × 2	Aramid: UHMWPE 10: 10	Aramid: UHMWPE 10: 10	0.9 mm

**Table 3:** Specification of resins.

Resin	Tensile Modulus	Bending Modulus	CTE
	/GPa	/GPa	/10^−6^·℃
VE (R806)	2.9	3.2	24
UP (191S)	3.8	3.4	135
EP (6349)	2.3	2.6	68

**Table 4:** Sample code symbols.

	VE (R806)	UP (191S)	EP(6349)
Aramid	A1	B1	C1
Aramid/Carbon	A2	B2	C2
Aramid/UHMWPE	A3	B3	C3

**Table 5:** Laser system.

Laser System	Parameter
Accuracy	0.034 mm
Point acquisition rate	23,040 points/s
Points per line	768
Line rate	30 Hz

**Table 6:** Configuration of feature points and feature distance.

	Pt1	Pt2	Pt3	Pt4	Pt5	Pt6	Pt7
Pt1							
Pt2	D1						
Pt3		D2			Symmetric		
Pt4	D4		D3				
Pt5	D5	D9					
Pt6			D10		D6		
Pt7	D8			D11		D7	

## Appendix A. Experimentally Determined Feature Distance of Reference model and Nine Helmets

	**cad**	**A1**	**A2**	**A3**	**B1**	**B2**	**B3**	**C1**	**C2**	**C3**
D1	204.842	206.803	206.525	207.418	205.238	207.202	208.397	206.226	205.985	207.428
D2	159.644	160.238	159.662	160.684	160.167	159.959	160.161	159.796	160.552	160.304
D3	121.785	119.088	118.804	118.335	119.293	119.041	119.173	118.689	119.800	119.181
D4	137.548	141.287	140.853	140.836	140.938	140.706	140.921	140.771	140.991	140.409
D5	204.843	203.732	204.061	203.892	203.856	202.865	203.513	204.203	204.142	204.285
D6	159.643	158.654	158.974	159.298	159.081	159.249	160.391	159.023	158.996	158.549
D7	121.778	118.478	118.022	117.972	118.233	118.098	117.628	117.545	118.059	117.864
D8	137.549	140.269	139.994	139.954	139.959	139.829	139.865	140.097	140.292	139.545
D9	266.751	273.218	272.438	277.264	272.792	271.219	279.388	274.154	275.594	277.388
D10	101.010	102.787	102.723	103.083	102.146	102.433	102.402	102.320	103.451	102.164
D11	106.908	108.148	107.411	107.629	107.338	107.336	107.670	107.263	108.049	106.998
Unit: mm										
